# Supplementary material for: Fundamental constraints in synchronous muscle limit superfast motor control in vertebrates
Source: eLife. 2017 Nov 22;6:e29425. doi: 10.7554/eLife.29425 (PMC5699865; doi:10.7554/eLife.29425)
Supplement: Supplementary file 1. [file elife-29425-supp1.docx]

# Supplementary Information

**Fundamental constraints in synchronous muscle limit superfast motor control in vertebrates,** Mead et al.

## 5

10

SUPPLEMENTARY FILE 1A. SR VESICLE CA2+ UPTAKE AND RELEASE. The SR vesicle

## Ca2+ uptake and release were analyzed fluorometrically in crude muscle homogenate. Nadir [Ca2+] is the [Ca2+] before initiating Ca2+ release assuming 0.2 g protein per g muscle wet weight. Intraspecific comparisons of SR vesicle Ca2+ uptake and release were significantly different between SFM and TA for zebra finch and bat.

Rate of Ca2+ uptake at 800 nM Ca2+ (µmol g ww-1 min-1) Rate of Ca2+ uptake at 800 nM Ca2+ (µmol g prot.-1 min-

| **ZF SFM**  (DTB) | **ZF**  (TA) | **Bat SFM**  (CT) | **Bat**  (TA) | **Toadfish SFM**  (swimbladder) | **Rat**  (EDL) | **Rat**  (SOL) |
| --- | --- | --- | --- | --- | --- | --- |
| 19.3 ± 1.8  * p<0.01 | 2.8 ± 0.2 | 13.4 ± 1.6  * p<0.01 | 1.6 ± 0.1 | 16.5 ± 0.9 | - | - |
| 96.5 ± 9.1  * p<0.01 | 13.9 ± 1.1 | 66.9 ± 8.0  * p<0.01 | 8.1 ± 0.5 | 82.3 ± 4.4 | 25.4±1.5 | 4.8 ± 0.3 |
| 13.6 ± 1.6  * p<0.01 | 2.1 ± 0.1 | 13.0 ± 1.4  * p<0.01 | 1.7 ± 0.1 | 12.1 ± 0.4 | - | - |
| 68.2 ± 8.0  * p<0.01 | 10.7 ± 0.5 | 64.9 ± 7.1  * p<0.01 | 8.7 ± 0.3 | 60.5 ± 1.9 | 13.7± 0.7 | 2.9 ± 0.1 |

1)

Rate of Ca2+ release at nadir Ca2+ (µmol g ww-1 min-1)

Rate of Ca2+ release at nadir Ca2+ (µmol g prot.-1 min-1)

## 15

1

SUPPLEMENTARY FILE 1B. ENSEMBLE/NCBI NAMES AND ACCESSION NUMBERS OF INVESTIGATED MYH GENES

## 20

| **Species** | **Ensemble/NCBI**  **Gene Name** | **Polypeptide**  **Accession #** | **Location** |
| --- | --- | --- | --- |
| **Zebra finch** | MYH13  Uncharacterized | ENSTGUG00000007387 HOZ747 | Chrom. 18  Chrom. 18 |
|  | Uncharacterized Uncharacterized | HOZ956 HOZ9U7 | Chrom. 18  Chrom. 18 |
|  | Uncharacterized Uncharacterized | HOZ816 HOZAJ6 | Chrom. 18  Chrom. 18 |
|  | Uncharacterized MYH7 | HOZ664 XP_012427262 | Chrom. 18  Chrom. 2 |
| **LB bat** | MYH13 MYH8 | ENSMLUG00000008752 ENSMLUG00000016365 | Scaff. GL40049 Scaff. GL40049 |
|  | MYH4 G1PUM0 | ENSMLUG00000016398 ENSMLUG00000016426 | Scaff. GL40049 Scaff. GL40049 |
|  | MYH2 MYH3 MYH7 | ENSMLUG00000016448 ENSMLUG00000016465 ENSMLUT00000017230 | Scaff. GL40049 Scaff. GL40049 Scaff. GL430064 |
| **Human** | MYH13 MYH8 | NP_003793 NP_002463 | Chrom. 17  Chrom. 17 |
|  | MYH4 MYH1 | NP_060003 NP_005954 | Chrom. 17  Chrom. 17 |
|  | MYH2 | NP_060004 | Chrom. 17 |
|  | MYH3 | NP_002461 | Chrom. 17 |
|  | MYH7 MYH6 | NP_000248 NP_002462 | Chrom. 14  Chrom. 14 |
|  | MYH9 MYH14 | NP_002464 NP_001070654.1 | Chrom. 19  Chrom. 20 |
|  | MYH15 | NP_055796 | Chrom. 3 |
| **Flying Fox** | MYH13 MYH8 | XP_011357427 XP_011357428 | Scaff 44  Scaff 44 |
|  | MYH4 MYH1 | ENSPVAT00000002931.1 XP_011357429 | Scaff 875  Scaff 875 |
|  | MYH2 MYH3 | XP_011357430 XP_011357263 | Scaff 875  Scaff 875 |
| **Python** | MYH7 LOC103063943 | XP_015745540 XP_007422923 | Scaff 236 |
|  | LOC103063706 LOC103063242 | XP_007422922 XP_015747018 | Scaff 236  Scaff 236 |
|  | LOC103063479 MYH7 | XP_007422921 XP_015745540 | Scaff 236  Scaff 4570 |
| **Torafugu** | MYH13 (M454)  Unchar. (M1878) | ENSTRUT00000013054.1 ENSTRUT00000038548.1 | Scaff 359  Scaff 139 |
| **Frog** | MYH13 (MyHC-270f) MYH4 (MyHC-270b) | ENSXETT00000035582.3 ENSXETT00000035510.2 | Scaff GL172891.1 Scaff GL172891.1 |
|  | Unchar. (MyHC-270a) Unchar. (MyHC-101d) | ENSXETT00000062626.1  Fgensh reconstruction | Scaff GL172891.1 Scaff GL172772.1 |
|  | MYH6 |  |  |

| **Chicken** | MYH13 MYH1A | ENSGALT00000051648.1 NP_001013414 | Chrom. 18  Chrom. 18 |
| --- | --- | --- | --- |
|  | MYH1B MYH1C | NP_989559 NP_001107181 | Chrom. 18  Chrom. 18 |
|  | MYH1D MYH1E | NP_001306233 NP_001013415 | Chrom. 18  Chrom. 18 |
|  | MYH1F MYH1G | NP_001305944 NP_001305945 | Chrom. 18  Chrom. 18 |

25 SUPPLEMENTARY FILE 1C. MRNA PRIMER DESIGN FOR DAUBENTON’S BAT SFM

Gene **Forward primer (5´- 3´) Reverse primer (5´- 3´)**

HPRT TGCTCGAGATGTGATGAAGG TCCCCTGTTGATTGGTCATT

MYH1 TGGCTGCCAGTGTATAATGC GCCAGAACCTTCCTCCTTCT

MYH2 CCCTCCCAAATACGACAAGA GTTATACACCGGCAGCCACT MYH3 CACCAAGAGGGTCATCCAGT GTTGTCGTTCCTCACGGTCT MYH4 TGCCTAGCATTGACGATCAG TCAGCAACTTCAGTGCCATC MYH8 CTACTCGGGCCTCTTCTGTG ATTCTCCGGTGATCAGGATG MYH13 GCATCGAGTGGGAGTTCATT GCTTCTGGAAGTTGGCAGAC

## 30

SUPPLEMENTARY FILE 1D. MRNA PRIMER DESIGN FOR ZEBRA FINCH SFM

| Gene | **Forward primer (5´- 3´)** | **Reverse primer (5´- 3´)** |
| --- | --- | --- |
| GAPDH | TGACCTGCCGTCTGGAAAA | CCATCAGCAGCAGCCTTCA |
| T gut MYH13 | GGCAGAACAGTTGCTGTCAA | ACGCAGAAGAGACCCGAGTA |
| T gut HOZ747 | AAGTGGATGATCTGGAAGGG | ATTGTATGCCTAGAAGTTGC |
| T gut HOZ956 | TGACAACGCCTATCAGTCCA | GCATTTTGCCTGATGCATGT |
| T gut HOZ9U7 | AGCCATGTTTGAGAAGATGT | CGAACATGTGGTGGTTGAAG |
| T gut HOZ816 | GGCATCTCTAATGAAAGAGA | TCAGCCCTTTCAGTCACCTC |
| T gut HOZAJ6 | CGTGAAGGAAGACCAAGTCT | GAGTAGGTGTAGATCATCCA |
| T gut HOZ664 | GGCAATCTTTGGAGAAGCTG | CAGTCACAGTTGTGTCATCG |

## 35
